# Enhanced Chemosensitivity by Targeting Nanog in Head and Neck Squamous Cell Carcinomas

**DOI:** 10.3390/ijms150914935

**Published:** 2014-08-25

**Authors:** Chuan-En Huang, Cheng-Chia Yu, Fang-Wei Hu, Ming-Yung Chou, Lo-Lin Tsai

**Affiliations:** 1 School of Dentistry, Chung Shan Medical University, No. 110, Sec. 1, Jianguo N. Rd., Taichung 40201, Taiwan; E-Mails: csmulab1@gmail.com (C.-E.H.); ccyu@csmu.edu.tw (C.-C.Y.); fang0989909009@gmail.com (F.-W.H.); myc@csmu.edu.tw(M.-Y.C.); 2 Department of Dentistry, Chung Shan Medical University Hospital, No. 110, Sec. 1, Jianguo N. Rd., Taichung 40201, Taiwan; 3 Institute of Oral Sciences, Chung Shan Medical University, No. 110, Sec. 1, Jianguo N. Rd., Taichung 40201, Taiwan

**Keywords:** head and neck squamous cell carcinomas, Nanog, cancer stem cells, chemosensitivity

## Abstract

Chemo-resistance is the major cause of high mortality in head and neck squamous cell carcinomas (HNSCC) in which HNSCC-derived cancer stem cells (CSCs) may be involved. Previously, we enriched a subpopulation of HNSCC-derived spheroid cells (SC) (HNSCC-SC) and identified Nanog as a CSCs marker. The aim of this study was to determine the role of Nanog in the chemosensitivity of HNSCC. The functional and clinicopathological studies of Nanog were investigated in HNSCC cells and specimens. Nanog expression was increased in HNSCC cell lines as compared to a normal oral epithelial cell line. Nanog upregulation in clinical tissues from HNSCC patients with recurrent and metastatic specimens relative to the mRNA levels in the samples from normal or primary tissues were examined. Targeting Nanog in HNSCC-SC significantly inhibited their tumorigenic and CSCs-like abilities and effectively increased the sensitivity of HNSCC-SC to chemotherapeutic drug cisplatin treatment. Targeting Nanog in HNSCC-SC showed a synergistic therapeutic effect with cisplatin. Our results suggest that targeting Nanog may have promising therapeutic potential for HNSCC.

## 1. Introduction

Head and neck squamous cell carcinomas (HNSCC) are a worldwide public health problem, and the incidence of HNSCC is increasing worldwide [1]. In spite of advanced improvement in both diagnosis and therapy in recent decades, the prognosis of HNSCC remains poor [2]. To increase the patient survival rate, investigations elucidating the mechanisms of tumorigenicity in HNSCC are urgently needed. Some studies have suggested that the subsets of cancer stem cells (CSCs) are key contributors to chemo-radio-resistance and are responsible for tumor progression, as well as recurrence after conventional therapy [2,3,4]. Development of CSC-specific targeting therapeutics could improve efficacies and increase the HNSCC patient survival rate and, thus, has become a prospective direction for cancer therapy.

Nanog is a key transcription factor that is involved in the maintenance of pluripotency and self-renewal in undifferentiated embryonic stem (ES) cells [5]. A recent study revealed that a short sequence in the well-conserved homeobox domain of Nanog was sufficient to induce pluripotency in Nanog-deficient somatic cells, indicating a crucial role of the homeobox domain in mediating the reprogramming ability of Nanog and that the transcriptional activity of Nanog might be dispensable [6]. Overexpression of Nanog has been reported by several groups in germ cell tumors, as well as other tumors, including breast, cervix, oral, kidney, prostate, lung, gastric, brain and ovarian cancer [7,8,9,10,11,12,13,14,15,16,17,18,19], though Schreiber and colleagues suggested that transcription of Nanog and Oct4 is unlikely to be a key determinant of CSCs properties [20]. Strong expression of Nanog is shown as an indicator of a poor prognosis for ovarian serous carcinoma, colorectal and breast cancer patients [21,22,23]. In HNSCC and lung adenocarcinoma, high expression of Nanog was associated with advanced cancer stage and shorter patient survival rate [8,17]. However, Nanog-mediated molecular mechanisms in HNSCC still remain to be elucidated.

In the present study, our results suggest that shRNA-mediated knockdown of Nanog can effectively block CSC-like properties and increase the sensitivity of HNSCC-CSCs to cisplatin treatment and induced cell apoptosis. This method has the potential to enhance the effects of conventional treatments for HNSCC patients.

## 2. Results and Discussion

### 2.1. The Upregulation of Nanog Expression in HNSCC (Head and Neck Squamous Cell Carcinomas Cell Lines and HNSCC Patients

**Figure 1:**
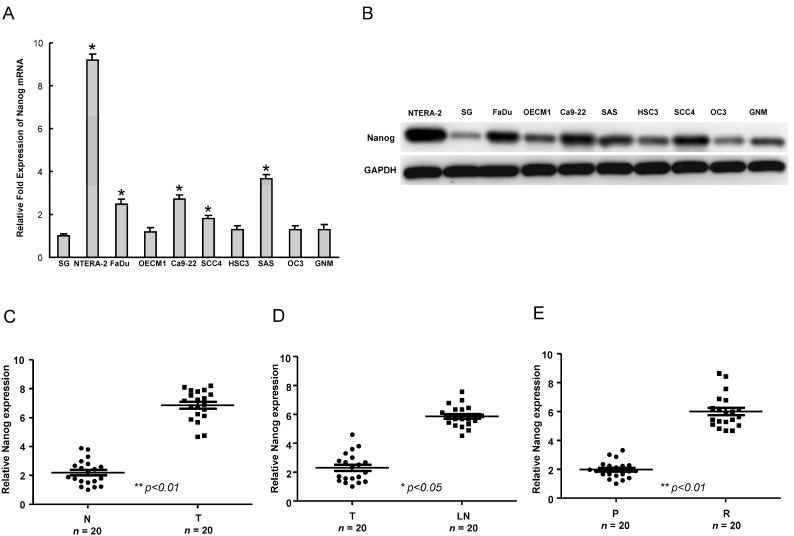
Determination of Nanog expression in head and neck squamous cell carcinomas (HNSCC) cells and HNSCC specimens. Nanog transcript (**A**) and protein (**B**) expression in eight HNSCC lines and one normal oral epithelial cell (SG) and one human embryonal carcinoma cell line (NTERA-2) were examined by real-time RT-PCR analysis and western blotting. The amount of GAPDH protein of different crude cell extracts was considered the loading control; (**C**) Quantitative RT-PCR analysis of Nanog expression levels in clinical specimens from a non-tumor region (N) or a tumor region (T); (**D**) Analysis of Nanog expression levels in primary and lymph node metastatic HNSCC tissues; and (**E**) Quantitative RT-PCR analysis of Nanog expression levels in clinical specimens from primary (P) and recurrent (R) HNSCC patients. * *p* < 0.05; ** *p* < 0.01.

To understand the expression of Nanog in HNSCC cell lines, the endogenous transcript and protein level of Nanog in eight established HNSCC cell lines, one normal oral epithelial cell line, SG, and one pluripotent human embryonal carcinoma cell line, NTERA-2, was examined by real-time RT-PCR analysis and western blot. As shown in Figure 1A,B, Nanog mRNA and protein were detectable in OSCC cell lines FaDu, Ca9-22, SAS and SCC4 HNSCCs. However, it was lower or undetectable in a normal oral epithelial cell line, SG (Figure 1A,B). To validate the significance of Nanog expression in clinical specimens, we collected paired samples of non-tumor (N), local tumor (T) and lymph node (LN) tissues from HNSCC patients and subjected these samples to real-time RT-PCR analysis. Compared with non-tumor samples from the same patient, the expression of Nanog was increased in the tumor samples (Figure 1C). A similar upregulation of Nanog was also observed in metastatic lymph nodes when compared with local tumors (T) (Figure 1D). We also compared the levels of these molecules between primary and recurrent lesions in HNSCC patient tissues. In line with our previous data, the level of Nanog expression was higher in recurrent HNSCC tumor samples, but lower in primary lesions (Figure 1E). These findings revealed an upregulated Nanog expression signature as a potential biomarker of HNSCC.

### 2.2. Reduction of CSCs (Cancer Stem Cells) Properties by Targeting Nanog

**Figure 2:**
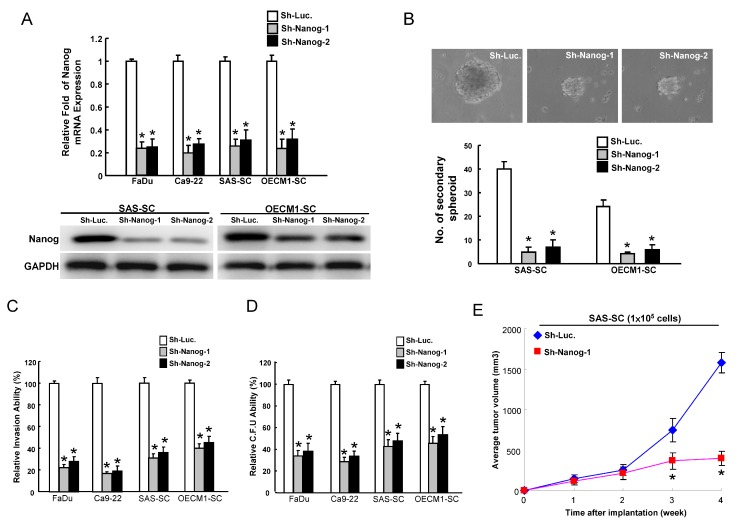
Targeting Nanog effectively abrogates the oncogenicity of HNSCC cells and HNSCC-spheroid cells (SCs). (**A**) The silencing effect of Nanog-shRNA in HNSCC cells and HNSCC-SCs was validated by real-time RT-PCR analysis and western blotting. Control and Nanog-knockdown HNSCC-SC were subjected to a secondary spheroid assay; (**B**) To elucidate the capability of cell invasiveness (**C**) and anchorage independent growth (**D**) of HNSCC-SC, HNSCC-SC with Nanog downregulation, single-cell suspension of HNSCC-SC infected with Nanog-specific shRNA or control sh-Luc lentivirus for three days were plated onto transwell and transwell coated with matrigel and soft agar, respectively, and analyzed as described in the Materials and Methods. Results are the means ± SD of triplicate samples from three experiments; and (**E**) Nude mice were subcutaneously injected with control and Nanog-knockdown SAS-SC, and the mice were monitored for six weeks of tumor development (*n* = 6). * *p* < 0.05.

CSCs are enriched in a population of cells that are capable of forming spheroid bodies under defined serum-free cultivation medium plus necessary growth factors according to individual solid tumors or cancers [24,25]. This cultivation condition also helps CSCs to maintain their undifferentiated state [24,25]. Previously, we enriched a subpopulation of spheroid cells (SCs) from SAS and OECM1 cells (SAS-SC and OECM1-SC) and upregulated Nanog in SAS-SC and OECM1-SC [17]. To further investigate whether Nanog could play a role in maintaining the CSC properties of HNSCC, the approach of loss-of-function of Nanog was first conducted. Downregulation of Nanog in HNSCC cell lines (FaDu and Ca9-22) and HNSCC-SCs (SAS-SC and OECM1-SC) was achieved by viral transduction with a lentiviral vector expressing small hairpin RNA (shRNA) targeting Nanog (sh-Nanog-1 and sh-Nanog-2), and lentiviral vector expressing shRNA against luciferase (sh-Luc) was used as a control. Real-time RT-PCR and immunoblotting analyses confirmed that lentivirus expressing both sh-Nanog-1 and sh-Nanog-2 markedly reduced the expression level of Nanog mRNA and protein in transduced HNSCC cell lines (FaDu and Ca9-22) and HNSCC-SC (SAS-SC and OECM1-SC)*.* Targeting Nanog in HNSCC-SC caused the loss of their secondary spheroid-forming ability (Figure 2B)*.* Downregulation of Nanog also inhibited the invasiveness (Figure 2C) and the number of soft agar colonies (Figure 2D) in HNSCC cell lines and HNSCC-SC. Furthermore, cells infected with sh-Nanog expressing lentivirus significantly slowed down the tumor growth mediated by SAS-SC (Figure 2E). Taken together, these findings demonstrated that silencing Nanog suppressed the CSC properties in HNSCC.

### 2.3. Silencing Nanog Enhances the Sensitivity of Chemotherapy and Decreases Drug-Resistant Markers

**Figure 3:**
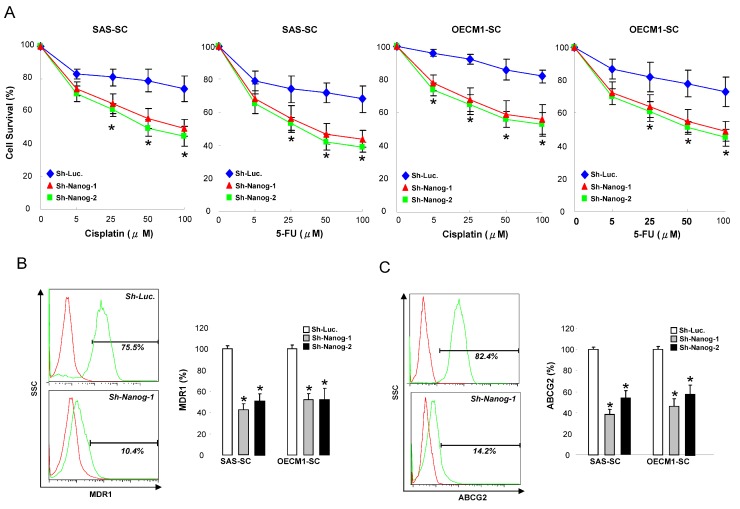
Nanog knockdown enhances the efficacy of cisplatin or fluorouracil (5-FU) chemotherapy in HNSCC-cancer stem cells (CSCs). (**A**) Control and Nanog knockdown HNSCC-SCs were subjected to treatment with different concentrations of cisplatin or 5-FU. Cell viability was determined by the methyle thiazol tetrazolium (MTT) assay; Expression drug-resistant markers MDR1 (**B**) and ABCG2 (**C**) in control and Nanog knockdown HNSCC-SC were measured by Fluorescence-activated cell sorting (FACS) analyses. * *p* < 0.05.

The observation of Nanog-mediated regulation of the CSC properties suggested its involvement in modulating the chemo-resistance of HNSCC-SC. Cell viability was measured to evaluate the sensitivity of the HNSCC-SC to chemotherapeutic drugs. Nanog-knockdown HNSCC-SCs were more sensitive to chemotherapeutic agents, including cisplatin and fluorouracil (5-FU), than the control cells (Figure 3A). The ABC (ATP-binding cassette) transporter family, including ABCB1 (also known as MDR1) and ABCG2, is associated with chemoresistance in cancers [26]. We therefore tested the effect of targeting on a panel of ABC transporters. Flow cytometry analysis of MDR1 and ABCG2 indicated that silencing decreased the percentage of cells with a high expression of MDR1-positive or ABCG2-positive cells (Figure 3B,C).

### 2.4. Co-Administration of Targeting Nanog with a Combination of Cisplatin Treatment Decreased Oncogenicity and Enhanced the Apoptosis Capability of HNSCC-SC

**Figure 4:**
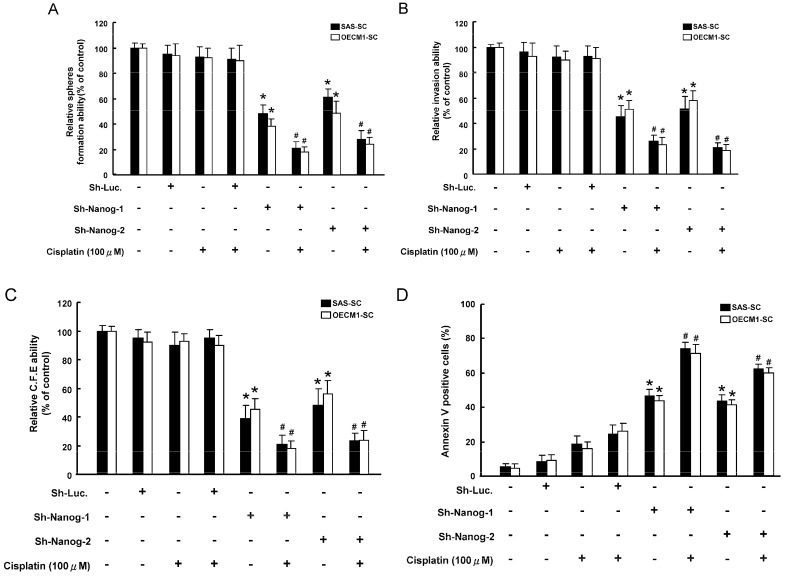
Synergistic effect of targeting Nanog combined with of cisplatin treatment decreased oncogenicity. Secondary spheroids formation (**A**); invasion ability (**B**) and colony-forming ability (**C**) were assessed in HNSCC-SCs treated with either sh-Nanog lentiviruses or cisplatin chemotherapy or both; and (**D**) Annexin V-positive cells were assessed in HNSCC-CSCs treated with either sh-Nanog lentiviruses or cisplatin chemotherapy or both. * *p* < 0.05 sh-Nanog-1 *vs*. sh-Luc; ^#^ *p* < 0.05 sh-Nanog-1 + Cisplatin *vs.* sh-Nanog-1 alone.

The combination Nanog-knockdown and cisplatin treatment showed a synergistic effect in abrogating self-renewal ability in HNSCC-SCs (Figure 4A). Single-cell suspension of Nanog-knockdown HNSCC-SCs treated with or without cisplatin treatment were used for analysis of their invasion/clonogenicity *in vitro*, as described in the Materials and Methods section. Treatment with cisplatin alone did not affect the invasion ability in HNSCC-SCs (Figure 4B). The combination of silencing Nanog and cisplatin treatment enhanced the efficacy of these treatments (Figure 4B). Meanwhile, similar synergistic effects of downregulation of Nanog and cisplatin treatment were also observed in a colony formation assay (Figure 4C). Similar synergistic effects were also observed in the mean number of Annexin V-positive cells in Nanog-knockdown HNSCC-SCs combined with cisplatin treatment (Figure 4D). Taken together, targeting Nanog exhibited a prominent therapeutic effect in enhancing the sensitivity of chemotherapy in HNSCC-CSCs.

### 2.5. Discussion

Nanog mRNA is present in pluripotent mouse and human stem cell lines and absent from differentiated cells [27]. Data revealing abnormal elevated expression levels of Nanog in several types of cancer stem cells suggest the importance and therapeutic potential of targeting these stemness regulators in cancers. Strong expression of Nanog is shown as an indicator of a poor prognosis for ovarian serous carcinoma, colorectal and breast cancer patients [21,22,23]. CD133^+^ or CD44^+^ cancer cells express significantly higher levels of Nanog in comparison to CD133^−^ or CD44^+^ ones, respectively [27,28,29]. On the other hand, Nanog induction in prostate cancer cell lines results in upregulation of CD133 and ALDH1 [30]. Ectopic overexpression of Nanog in prostate cancer cells enhanced clonal growth and tumor regenerative capacity [30], and the activation of the embryonic *NANOG* gene caused a subpopulation of colorectal cancer cell to adopt a stem-like phenotype [30]. Knockdown of Nanog impeded cell proliferation, migration and invasion. Overexpression of Nanog has been associated with chemoresistance in HNSCCs [31]. Herein, we found that Nanog expression was increased in HNSCC cell lines and specimens (Figure 1). Notably, targeting Nanog expression significantly suppressed the CSCs properties and inhibited the endogenous expression of MDR1 and ABCG2 in HNSCC-SCs. Importantly, downregulation of Nanog ameliorated the chemoresistance of HNSCC-SCs to cisplatin, synergistically increasing the efficacy of cisplatin. These results demonstrated the potential for the development of novel strategies to suppress the progression of HNSCCs, while improving chemosensitivity.

Epithelial mesenchymal transition (EMT) is a de-differentiation program that converts adherent epithelial cells into individual migratory cells. EMT is thought to be a key step in the induction of tumor malignancy, oncogenic progression and cancer metastasis [32]. The correlation between Nanog overexpression and advanced stage of cancer or metastatic incidence indicates a crucial role of Nanog in metastasis. Stable knockdown of Nanog in ovarian cancer cell lines resulted in increased E-cadherin, FOXO1, FOXO3a, FOXJ1 and FOXB1 mRNA levels, whereas ectopic Nanog overexpression decreased them [33]. It is further claimed that Nanog-mediated cell migration and invasion involved its regulation of FOXJ1 and E-cadherin [33]. A further understanding on the regulatory networks between Nanog and EMT may update our current knowledge on the development of therapeutic treatments for HNSCC in the future.

MicroRNAs (miRNAs) may function as either oncogenes or tumor suppressors in cancer progression [34]. Recent studies have shown that specific miRNA expression profiles may predict prognosis and disease recurrence in HNSCC [35,36]. With the increasing awareness of the importance of miRNAs in tumorigenicity, accumulating evidence has been reported supporting the involvement of miRNAs in CSC properties [34,35,36,37]. Recently, Iliopoulos and colleagues reported that miR200b regulates CSCs properties through directly targeting Suz12, a subunit of a polycomb repressor complex [38]. For example, miR200a reduced the stem-like state and epithelial-mesenchymal transition through targeting ZEB2 and β-catenin signalings in nasopharyngeal carcinoma cells [39]. miR34a inhibits prostate tumor regeneration and metastasis through direct repression of the CD44 prostate CSC marker [40]. miR200c has also been demonstrated to attenuate tumor growth and metastasis of the ALDH^+^/CD44^+^ HNSCC-CSCs [41]. It is therefore possible that Nanog might also be regulated by miRNAs in HNSCC-CSCs. Further research effort is needed in this area. 

## 3. Experimental Section

### 3.1. HNSCC Tissues Acquirement and Preparation

Surgical tissue specimens from HNSCC patients were collected after obtaining written informed consent, and this study was approved by The Institutional Review Board in Chung Shan Medical University Hospital (CSMUH No.: CS10249). Human primary HNSCC carcinoma (T) tissue, normal paired noncancerous matched tissues (N), as well as available lymph node metastatic lesions (M) were obtained from surgical specimens sent to the pathology lab for frozen section diagnosis. Tumor tissues were microscopically screened to have >70% of their areas occupied by tumor cells; the remaining specimens (tumor, normal counterpart and lymph node metastatic lesions) were snap frozen in liquid nitrogen and stored at −80 °C for real-time reverse transcription-PCR (qRT-PCR).

### 3.2. Quantitative Real-Time Reverse-Transcriptase (RT)-PCR

Real-time RT-PCR was performed as previously described (7–10). Briefly, total RNA (1 μg) of each sample was reverse-transcribed in a 20-μL reaction using 0.5 μg oligo (dT) and 200 U Superscript II RT (Invitrogen, Carlsbad, CA, USA). The amplification was carried out in a total volume of 20 μL containing 0.5 μM of each primer, 4 mM MgCl_2_, 2 μL LightCyclerTM–FastStart DNA Master SYBR Green I (Roche Molecular Systems, Alameda, CA, USA) and 2 μL of 1:10 diluted cDNA. PCR reactions were prepared in duplicate and heated to 95 °C for 10 min, followed by 40 cycles of denaturation at 95 °C for 10 s, annealing at 55 °C for 5 s and extension at 72 °C for 20 s. Standard curves (cycle threshold values *vs.* template concentration) were prepared for each target gene and for the endogenous reference (GAPDH) in each sample. Quantification of unknown samples was performed using LightCycler Relative Quantification Software Version 3.3 (Roche Molecular Systems, Alameda, CA, USA). Primer sequences are listed in Table 1.

**Table 1:** The sequences of the primers for quantitative RT-PCR.

Gene (Accession No.)	Primer Sequence (5' to 3')	Product Size (bp)	Tm (°C)
Nanog (NM_024865)	F: ATTCAGGACAGCCCTGATTCTTC R: TTTTTGCGACACTCTTCTCTGC	76	60
GAPDH (NM_002046)	F: CATCATCCCTGCCTCTACTG R: GCCTGCTTCACCACCTTC	180	60

### 3.3. Construction of Lentiviral-Mediated RNAi for Silencing Nanog

The pLV-RNAi vector, which co-expressed for GFP protein in infected host cells, was purchased from Biosettia Inc. (Biosettia, San Diego, CA, USA). The method of cloning the double-stranded shRNA sequence was described in the manufacturer’s protocol. Lentiviral vectors expressing shRNA that targets human Nanog were synthesized and cloned into pLVRNAi to generate a lentiviral expression vector. sh-Luc: 5'-CCGGACTTACGCTGAGTACTTCGAACTCGAGTTCGAAGTACTCAGCGTAAGTTTTTTG-3'; sh-Nanog-1: 5'-AAAAGCATCCGACTGTAAAGAATTTGGATCCAAATTCTTTACAGTCGGATGC-3'; sh-Nanog-2: 5'-AAAAGCTGTGTGTACTCAATGATTTGGATCCAAATCATTGAGTACACACAGC-3' was utilized for an experimental control. Lentivirus production was performed as above. Stable pLV-RNAi-expressed HNSCC cell lines were further purified by cell sorting with GFP-positive cells. 

### 3.4. Assays for Cell Proliferation

An MTT assay kit (MTT Sigma-Aldrich, St. Louis, MO, USA) was used to analyze the cell proliferation. Specifically, 1 × 10^3^ cells were seeded in each well of a 24-well plate, and then, 10 μL of MTT solution were added to the cells, which were then incubated at 37 °C for 3 h. The supernatant was removed, and 200 μL of Dimethyl sulfoxide (DMSO) were added directly to the cells. The MTT color reaction was analyzed using a microplate reader set at A560 nm.

### 3.5. Identification of Cell Phenotypic Markers by Fluorescence-Activated Cell Sorting (FACS)

Cells were used for phenotypic marker identification by flow cytometry. One-hundred thousand cells were resuspended in 100 µL phosphate-buffered saline (PBS) and incubated with primary MDR1 and ABCG2 antibodies at 4 °C for 1 h with 1:100 dilutions. The labeled cells were suspended in 100 µL PBS with 1 µL goat anti-mouse IgG conjugated with fluorescein isothiocyanate (FITC) (Chemicon, Temicula, CA, USA) at 4 °C for 1 h, then examined with a FACSCalibur apparatus (Becton Dickinson, La Jolla, CA, USA).

### 3.6. In Vitro Cell Invasion Assay

*In vitro* cell invasion analysis was conducted as described previously [41].

### 3.7. Tumorsphere-Forming Assay

Tumor cells were dissociated and cultured as tumorspheres in modified Dulbecco’s Modified Eagle Medium (DMEM)/F-12 plus N2 supplement (Invitrogen, Carlsbad, CA, USA), 10 ng/mL epidermal growth factor (EGF, Invitrogen), 10 ng/mL basic fibroblast growth factor (bFGF, Invitrogen) and penicillin/streptomycin at 10^3^ live cells/low-attachment six-well plate (Corning Inc., Corning, NY, USA), and the medium was changed every other day until the tumor sphere formation was observed in about 2 weeks. For serial passage of spheroid cells, single cells were obtained from accutase-treated spheroids, and the cell density of passage was 1000 cells/mL in the serum-free medium, as described above [4].

### 3.8. Soft Agar Colony Forming Assay

Each well (35 mm) of a six-well culture dish was coated with 2 mL of bottom agar (Sigma-Aldrich Co., St. Louis, MO, USA) mixture (DMEM, 10% (*v*/*v*) fetal calf serum (FCS), 0.6% (*w*/*v*) agar). After the bottom layer was solidified, 2 mL of top agar-medium mixture (DMEM, 10% (*v*/*v*) FCS, 0.3% (*w*/*v*) agar) containing 2 × 10^4^ cells were added, and the dishes were incubated at 37 °C for 4 weeks. Plates were stained with 0.005% Crystal Violet; then, the colonies were counted. The total number of colonies with a diameter ≥100 μm was counted over five fields per well for a total of 15 fields in triplicate experiments [4].

### 3.9. Subcutaneous Xenografts in Nude Mice

All of the animal practices in this study were approved and in accordance with the Institutional Animal Care and Use Committee (IACUC) of Chung Shan Medical University, Taichung, Taiwan. One million HNSCC cells mixed with Matrigel (BD bioscience, San Diego, CA, USA) (1:1) were injected subcutaneously into BALB/c nude mice (6–8 weeks). Tumor volume (TV) was calculated using the following formula: TV (mm^3^) = (length × width^2^)/2 [42].

### 3.10. Apoptotic Assay

Apoptotic cells were detected with an Annexin V-APC kit (Calbiochem, Darmstadt, Germany) according to the manufacturer’s guidelines. After staining, the cells incubated with 20 μg/mL propidium iodide (PI) were analyzed by FACS Calibur apparatus (Becton Dickinson, San Diego, CA, USA).

### 3.11. Statistical Analysis

The statistical package of Social Sciences software (Version 13.0) (SPSS, Inc., Chicago, IL, USA) was used for statistical analysis. The Student’s *t*-test was used to determine statistical significance of the differences between experimental groups; *p-*values less than 0.05 were considered statistically significant. The level of statistical significance was set at 0.05 for all tests.

## 4. Conclusions

The present report showed that targeting Nanog was a key mechanism for reducing CSC-like and chemoresistant properties in HNSCC. The promising therapeutic prospect of targeting Nanog for treating HNSC-CSCs may render it a potential approach to improving current cancer treatments, especially for those tumors that have developed a resistance to conventional therapeutic methods.
